# Contrecoup injury of the prefronto-thalamic tract in a patient with mild traumatic brain injury: A case report

**DOI:** 10.1097/MD.0000000000021601

**Published:** 2020-08-07

**Authors:** Sung Ho Jang, Young Hyeon Kwon, Sung Jun Lee

**Affiliations:** Department of Physical Medicine and Rehabilitation, College of Medicine, Yeungnam University 317-1, Daemyungdong, Namku, Daegu, Republic of Korea.

**Keywords:** diffusion tensor tractography, prefrontal cortex, prefronto-thalamic tract, traumatic brain injury

## Abstract

**Rationale:**

We report on a patient with mild traumatic brain injury (TBI) with contrecoup injury of the prefronto-thalamic tract (PTT), as demonstrated by diffusion tensor tractography (DTT).

**Patient concerns:**

A 62-year-old female patient suffered a head trauma after falling backward. While working at a height of 85cm above the floor, she fell backward and struck the occipital area of her head on the ground. The patient experienced cognitive dysfunction and depressive mood after the head trauma.

**Diagnoses:**

The patient was diagnosed as mild TBI due to falling backward.

**Interventions:**

Clinical evaluation of her brain was performed at 2 months after onset.

**Outcomes:**

DTT at 2 months after onset revealed narrowings in the right ventrolateral and both orbitofrontal PTTs, whereas both the dorsolateral and left ventrolateral PTTs were not reconstructed.

**Lessons:**

Injuries of the PTTs associated with a contrecoup brain injury were demonstrated in a patient with mild TBI.

## 1 Introduction

The prefrontal cortex is involved in various cognitive functions including memory, attention, decision making, execution, behavior inhibition, and motivation.^[1]^ The prefrontal cortex (PFC) consists of 4 regions: dorsolateral, ventrolateral, orbitofrontal, and medial.^[1]^ The PFC is connected with the thalamic mediodorsal nucleus through the prefronto-thalamic tract (PTT).^[2–4]^ The recently developed imaging method of diffusion tensor tractography (DTT), which permits tract reconstruction from diffusion tensor imaging (DTI) data, enables the visualization and localization of the PTT.^[2–4]^ A few studies using DTT have demonstrated PTT injury in traumatic brain injury (TBI) patients.^[5,6]^

Brain injury can be classified as coup or countrecoup types. A coup brain injury usually occurs when a moving object directly strikes the head, consequently causing the brain to collide with the skull; in contrast, a contrecoup brain injury usually occurs on the side opposite the side of the original impact, especially when a moving head strikes a stationary object.^[7,8]^

In the current study, we report on a patient with mild TBI exhibiting injury of the PTTs from a contrecoup brain injury as demonstrated by DTT.

## 2 Case report

**Figure 1:**
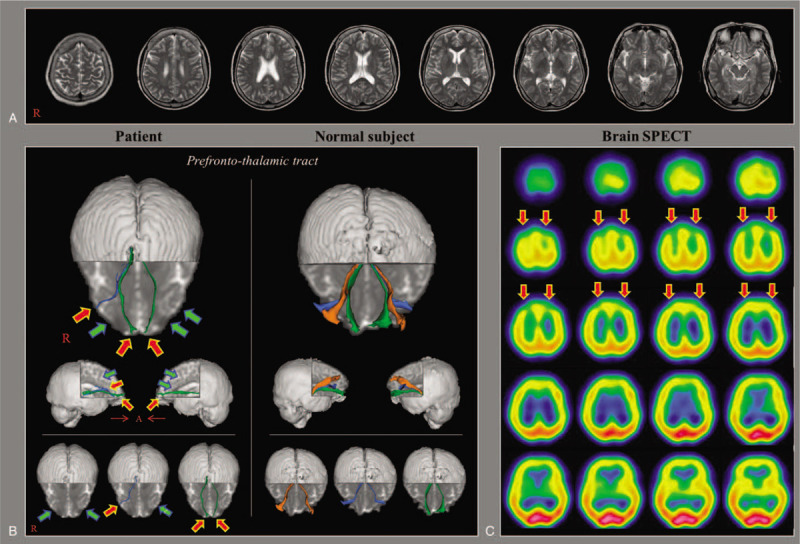
(A) T2-weighted brain magnetic resonance images at 2 months after injury onset show no abnormal lesion. (B) Imaging results for the prefronto-thalamic tracts (PTTs) on diffusion tensor tractography at 2 months after onset compared to that for a normal subject (60-year-old male). Narrowings (red arrows) are observed in the right ventrolateral and both orbitofrontal PTTs, and both the dorsolateral PTTs and the left ventrolateral PTT were not reconstructed (green arows). (C) Brain SPECT (Tc-99m ECD) images show reduction of perfusion in both the frontal lobes, especially in both prefrontal lobes (red arrows).

A 62-year-old female patient suffered from head trauma resulting from falling backward. While working at a height of 85 cm above the floor and adjacent to a device for bathing old people, she fell backward and struck the occipital area of her head against the ground. The patient mentioned that she experienced loss of consciousness and post-traumatic amnesia for approximately 1 minute after the accident.^[9]^ After the head trauma, she experienced cognitive dysfunction and a depressive mood. At 2 months after the head trauma, she came to the rehabilitation department of our university hospital for evaluation of her brain. No specific lesion was observed on brain magnetic resonance imaging (MRI) that included T1-weighted, T2-weighted, and fluid attenuated inversion recovery [FLAIR] images (Fig. 1-A). In contrast, brain single-photon emission computed tomography (SPECT) using technetium-99m ethyl cysteinate dimer (Tc-99m ECD) showed a reduction of perfusion in both frontal lobes, especially in both prefrontal lobes (Fig. 1-C). In addition, she exhibited neuropsychological impairment on various neuropsychological tests as follows: decreased general cognition (IQ) on Wechsler Adult Intelligence Scale = 74 (percentile 4); impaired memory on Memory Assessment Scale (MAS), global memory = 70 (percentile 2); and moderate depression on Beck Depression Inventory-II (BDI-II) = 23 (full score = 63).^[10–12]^ The patient provided signed, informed consent, and the study protocol was approved by our university hospital's institutional review board.

### 2.1 Diffusion tensor imaging

The DTI data were acquired 10 days after TBI onset by using a 6-channel head coil on a 1.5 T Philips Gyroscan Intera (Philips, Best, Netherlands) with single-shot echo-planar imaging. For each of the 32 non-collinear diffusion sensitizing gradients, 67 contiguous slices were acquired parallel to the anterior commissure–posterior commissure line. Imaging parameters were as follows: acquisition matrix = 96 × 96, reconstructed to matrix = 192 × 192, field of view = 240 mm × 240 mm, TR = 10,398 ms, TE = 72 ms, parallel imaging reduction factor (SENSE factor) = 2, echo-planar imaging factor = 59 and b = 1000 s/mm^2^, NEX = 1, and slice thickness = 2.5 mm.

Diffusion-weighted imaging data were analyzed by using software from the Oxford Centre for Functional Magnetic Resonance Imaging of the Brain (FMRIB) Software Library (FSL version 5.0; www.fmrib.ox.ac.uk/fsl). Affine multi-scale two-dimensional registration was used for correction of head motion and eddy current-induced image distortion. Fiber tracking was performed by using a probabilistic tractography method based on a multiple tensor model and using tractography routines implemented in FMRIB Diffusion (0.5 mm step lengths, 5000 streamline samples, curvature thresholds = 0.2).

To reconstruct the 6 PTTs (*i.e*., dorsolateral, ventrolateral, and orbitofrontal PTTs in both hemispheres) seed regions of interest (ROIs) were placed on the mediodorsal nucleus, and target ROIs were on the dorsolateral PFCs, the ventrolateral PFCs, and the orbitofrontal PFCs, respectively.^[4]^ The target ROIs were as follows: dorsolateral PFCs — Brodmann areas (BAs) 8, 9, and 46 on the coronal b0 image; ventrolateral PFCs — BAs 44, 45, and 47 on the coronal b0 image; and orbitofrontal PFCs — BAs 10, 11, and 13 on the axial image. Out of 5000 samples generated from a seed voxel, results were visualized at a threshold of 2 streamlines through each voxel for analysis.

On DTT, narrowings were observed in the right ventrolateral PTT and in both orbitofrontal PTTs, whereas both the dorsolateral PTTs and the left ventrolateral PTT were not reconstructed by DTT (Fig. 1-B).

## 3 Discussion

The contrecoup injury is mainly caused by traffic accident, fall down, and assault.^[13]^ The pathophysiological mechanism of brain injury by the contrecoup injury has not clearly elucidated, however, the contrecoup injury is known to be related to a higher mortality rate compared to the coup injury.^[8,14]^ In addition, the contrecoup injury can cause focal or diffuse brain white matter changes in the area of the frontal, temporal, and occipital depending on the location, hence, accurate diagnosis of this injury is clinically important.^[13,15]^ Although conventional brain computed tomography (CT) and MRI have been generally used to diagnose brain injury, these are known to have a limitation in precise detection of axonal lesions in the brain white matter following TBI.^[16,17]^ By contrast, DTI, which is recently introduced, can detect subtle changes in brain white matter which are not detectable on conventional brain CT and MRI.^[16,17]^ Especially, DTT, which is derived from DTI, has an unique advantage to detect axonal lesions of the various neural tracts in the brain in patients with TBI.^[18,19]^

In the current study, by using DTT, we undertook reconstruction of 6 PTTs (the dorsolateral, ventrolateral, and orbitofrontal PTTs in both hemispheres), and neural injuries in all 6 PTTs were detected in a patient with mild TBI. The main functions of the PTTs are as follows: the dorsolateral PTT – working memory and depression, and ventrolateral PTT – deliberation of decision making and goal-directed behavior, the orbitofrontal PTT – emotional control and inhibitory control of behavior.^[4,5,20–24]^ Among the 6 neural tracts, one neural tract showed partial injury (narrowing in the right ventrolateral PTT and both oribitofrontal PTTs) while the other five neural tracts showed severe injuries (non-reconstruction in both dorsolateral PTTs and the left ventrolateral PTT). The patient showed severe neuropsychological dysfunction with decreased general cognition (decreased IQ score), memory impairment (decreased MAS score), and moderate depression on BDI-II.^[10–12]^ Our DTT results revealing neural injuries of the PTTs connecting the prefrontal cortex and the thalamic mediodorsal nuclei are consistent with the results of neuropsychological testing related to the prefrontal lobe.^[4,5,20–24]^ In addition, the observation of decreased perfusion in both frontal lobes on brain SPECT appears to be evidence that supports the presence of PTT injuries. Because the PPTs and frontal lobes are located on the side opposite the occipital area (*i.e*., the head region that struck the ground) we suggest that the PPTs were injured as the result of a contrecoup brain injury.

In conclusion, by applying DTT, injuries of the PTTs resulting from a contrecoup brain injury were demonstrated in a patient with mild TBI. Our results suggest that evaluation of the PTTs via DTT in patients who present prefrontal lobe-related neuropsychological abnormality following brain injury would be helpful in elucidating their neurological state. However, some limitations of this study should be considered. Because it is based on a single case report, this study is limited; thus, we suggest that further studies involving large numbers of patients should be encouraged. Although DTT is a good anatomic imaging tool that can demonstrate gross fiber architecture, the presence of kissing fibers in regions of fiber complexity or the detection of false positive fiber trajectories may result in underestimation or overestimation of the completeness of a neural tract.^[25]^

## Author contributions

Sung Ho Jang, study concept and design, manuscript development, wrting, and funding.

Young Hyeon Kwon, study support, revising the image.

Sung Jun Lee, critical revision of manuscript for intellectual content, drafting/revising the image.
